# Toward adaptive control power sharing and bus voltage regulation for DC microgrids

**DOI:** 10.1038/s41598-026-47219-w

**Published:** 2026-04-24

**Authors:** Mohamed A. Mosbah, Ahmed Abokhalil, Khairy Sayed

**Affiliations:** 1 Faculty of Technology and Education, Sohag University, Sohag, Egypt 82524; 2 University of Sharjah, Sharjah, United Arab Emirates; 3 Faculty of Engineering, Sohag University, Sohag, 82524 Egypt

**Keywords:** Distribution resources, Droop control, Adaptive droop control, Distributed energy, Low voltage, Power sharing, Energy science and technology, Engineering, Mathematics and computing

## Abstract

Decentralized control for the DC microgrid has made extensive use of droop-based control. But correct power sharing and necessary voltage management are both impossible with traditional droop control, which results in circulating current. A novel adaptive control method that achieves precise power sharing and suitable voltage regulation based on the loading condition is presented in this paper for multiple converter DC microgrid applications. Since the output currents of the distributed power sharing units are far lower than the top limits, the accuracy of the power sharing procedure is not a problem under light load conditions. Because the output currents of the scattered producing units increase in proportion to the load, precise current sharing is required during high load situations. As the load level rises, the recommended control strategy improves the equivalent droop gains and offers precise current sharing. The innovative and novel adaptive droop controller has reduced the variance in load current sharing by utilizing the principal current sharing loops to update the droop settings and verifying them exist. To eliminate the bus voltage fluctuation in the DC microgrids, the second loop also shifted the droop lines. A variety of input voltages and load resistances are used to test the proposed method. The performance and stability of the suggested approach are assessed in this work using a linearized model, and the findings are confirmed using a suitable model created in MATLAB/SIMULINK and with the principles of real-time simulation.

## Introduction

 Due to its many advantages over AC microgrids, including increased efficiency, stability, and dependability, DC microgrids are becoming increasingly popular in various power DS. To increase overall efficiency and dependability, It takes a lot of power electronics-based interface devices to integrate DC-based energy sources like photovoltaics and storage units. The future expansion of DC microgrids is suggested by the elimination of reactive power, less transmission loss, and lower costs due to the removal of power conversion operations. The development of DC microgrids at the distribution level is ongoing. In essence, a DC microgrid paradigm that does away with unnecessary conversion phases is supported by the growing number of DC-based renewable energy technologies, such as PV solar, electric vehicles hybrids, storage batteries, and ultracapacitors. Electronic DC loads make up a large number of the new loads^1^. A microgrid consists of several converters connected in parallel that share current among several dispersed resources at a single DC bus. Today, DC microgrids are a feasible solution for satisfying load demands and covertly growing DC applications because of the potential benefits of DC systems over AC technology. Apart from the harmonic issues brought on by the large number of nonlinear loads in a power distribution network, DC microgrids are thought to be a better option than AC microgrids. Much work has been done on the design, control, and communication of MGs, but much more is required for developing distributed and autonomous MGs with multiple distributed energy^2,3^. One of the key problems for parallel converters is the circulating current. It decreases the power quality and interferes with the system’s stable operation. A new circulating current suppression technique is suggested for the parallel current source converter without a communication line to lower the circulating current of the converter and lessen harmonic pollution to the power grid. By altering the outside appearance of the parallel modules, this technique can achieve current sharing between them and so reduce the circulating current among the parallel current source converters^4^.

A decentralized control method known as “Droop Control” is implemented locally on each dispatchable distributed generating unit. Without relying on data from loads or other DGs, it makes autonomous control decisions based on local measurements. This allows dispatchable DG units to respond to transients and load dynamics in a timely and efficient manner, which is an essential capability, particularly for islanded MGs to maintain a stable operation with high reliability and plug-and-play functionality^5^. Droop settings are established in conventional droop-controlled island MGs according to the power ratings of the participating DGs to distribute the power demand proportionately to their power ratings^6^. Although this droop setting concept is straightforward, voltage dips across the transmission line impedances have a significant impact on the effectiveness of droop management. The accuracy with which MGs share reactive power in AC MGs and current in DC MGs is hampered by these voltage drops, which effectively result in differential bus voltages across the MGs’ common distribution line. Therefore, more effective droop setting design techniques are needed to enhance the performance of the conventional droop control strategy^7,8^. The converter’s output voltage mismatch is reduced via a hierarchical control algorithm that employs the droop technique. Methods that ignore the impact of connecting line impedances have also been presented^9^. To maintain power sharing and smooth the output voltage, a distributed secondary control technique based on average current and voltage management and circulating current minimization is employed^10^. To further reduce line voltage, drop and power losses, an extra current feedback loop is added to adjust the micro-grid reference voltage under overload conditions. To implement the suggested distributed control, information is exchanged with the appropriate controllers simultaneously via the traditional communication lines^11^.

As load variables or grid impedance change, adaptive droop control, an effective droop control technique for power systems like DC or AC microgrids, automatically modifies droop parameters^12^. Instead of using set parameters as in classic droop control, adaptive approaches adjust the droop gain, virtual impedance, or other control parameters in real-time to enhance power sharing, voltage regulation, and overall system stability. Particularly in intricate or dynamic settings, this enables more accurate management and can facilitate smooth transitions between grid-connected and islanded operating modes^13^.

**Fig. 1:**
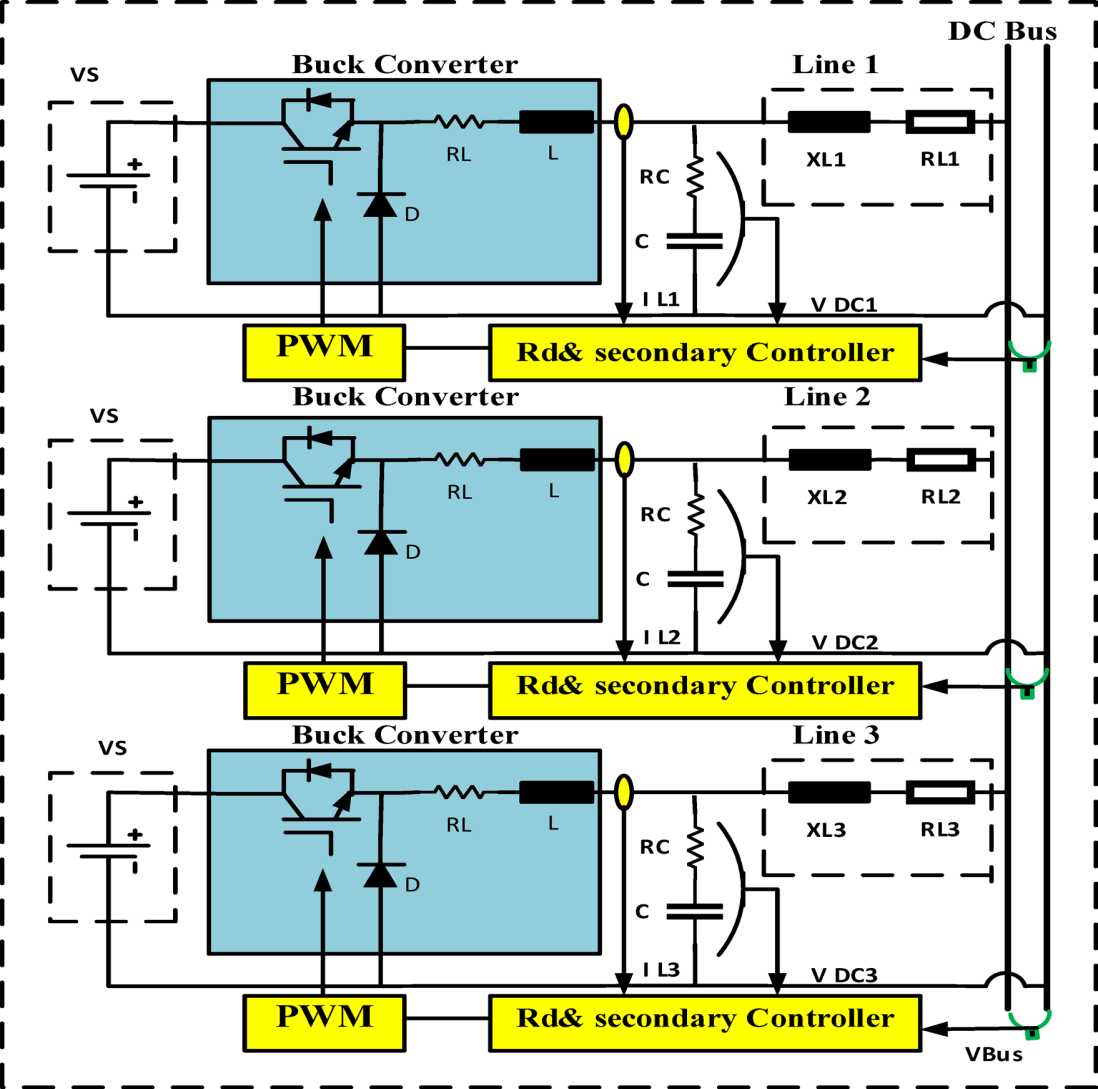
Schematic diagram for adaptive droop control.

Using locally monitored bus voltages, non-communication-based control methods allow power sharing across distributed energy resources^14^. They have many benefits, including low cost, high modularity, flexibility, expandability, and dependability, as well as ease of installation. Therefore, control methods that do not depend on communication systems are more appropriate for usage in some of the future DC microgrids that have many DERs spread out geographically^15^. Droop control solutions that do not rely on communication include conventional droop control, improved droop control, DC bus signals, and mode-adaptive droop control systems^16^. Providing efficient and appropriate output voltage regulation values for the current shared across the converters is the fundamental goal of DC microgrid control^17^. The effectiveness of traditional droop is significantly impacted by line impedance. There are two modes of operation for the droop control method: linear and non-linear. Because line impedance has a detrimental influence on the linear droop mechanism, the non-linear droop mechanism was selected^18^. It has been observed that the trade-off between voltage regulation and current sharing is delayed by the non-linear characteristics of droop control. The distributed control technique may perform adaptive droop control at the secondary and primary control levels due to enhanced communication and faster data transmission between converters. The droop coefficient can be softly adjusted under different loading scenarios thanks to the adaptive droop gain technique^19^. To provide voltage and impedance correction terms, the voltage and current regulators are installed at the secondary level. The droop parameters were examined online and adjusted using the principal current sharing loops to lessen the variance in the load current sharing^20^. Figure 1 illustrates the schematic diagram of the proposed adaptive droop control strategy. This approach ensures accurate power sharing among converters while maintaining proper voltage regulation in the DC microgrid.

To reduce the impact of line resistance and increase the precision of load sharing, a secondary controller is suggested^21^. Depending on the converter current, the secondary controller modifies the droop controller voltage setpoint^22–24^. Accurate load sharing is achieved by both suggested approaches. The proposed algorithm is tested with various input voltages and load resistances. to demonstrate the suggested methods efficacy in contrast to primary droop control^25–30^. To verify the precision and efficacy of the recommended control strategy, the pertinent model is built in MATLAB/SIMULINK^31–39^.

To highlight the limitations of existing strategies and the position contribution of this work, Table 1 provides a comparative overview of various droop-based control methods for DC microgrids. The comparison emphasizes their main advantages, shortcomings, and the remaining research gaps. While conventional and modified droop approaches offer simplicity, they fail to ensure accurate power sharing and voltage stability under dynamic loading. Hierarchical methods improve performance but at the cost of added communication and complexity. This underlines the necessity for an adaptive, decentralized solution capable of real-time parameter adjustment without relying on communication links.

**Table 1:** Comparison of droop-based control strategies for DC microgrids.

Control method	Advantages	Limitations	Remarks/research gap
Conventional droop control	Simple, decentralized, no communication needed	Inaccurate current sharing, bus voltage deviation, circulating current issues	Not suitable for precise load sharing under varying load conditions
Fixed gain modified droop	Improved current sharing compared to conventional droop	Gains tuned for specific operating conditions; poor adaptability under dynamic load variations	Limited scalability; requires re-tuning for changing system conditions
Secondary/hierarchical control	Enhances voltage restoration and improves sharing accuracy	Relies on communication links, increases complexity and cost, slower dynamic response	Vulnerable to communication delays/failures; undermines fully decentralized nature
Nonlinear/adaptive droop (static)	Better accuracy in current distribution through nonlinear gain adjustment	Gains predetermined offline; limited ability to cope with real-time disturbances	Insufficient adaptability to unpredictable load dynamics
Proposed adaptive droop control	Real-time adjustment of droop gains; precise current sharing under heavy loads; maintains voltage stability	Implementation complexity higher than conventional droop, but avoids communication dependency	Addresses circulating current and voltage deviation simultaneously; ensures robustness

The comparison in Table 1 makes it evident that no existing droop-based strategy fully addresses the dual challenge of precise current sharing and reliable bus voltage regulation in multi-converter DC microgrids. Conventional droop control and its static modifications lack adaptability to varying load conditions, while hierarchical and communication-based approaches compromise scalability and robustness. In contrast, the proposed adaptive droop controller directly responds to load variations by dynamically tuning droop gains in real time, thereby minimizing circulating currents and ensuring both accurate power sharing and voltage stability. This adaptive mechanism effectively bridges the identified research gap, offering a practical and decentralized solution for next-generation DC microgrid applications.

Although droop-based decentralized control has been widely adopted in DC microgrids due to its simplicity and scalability, it suffers from critical limitations in ensuring accurate power sharing and voltage regulation. Traditional droop control cannot simultaneously guarantee proper current distribution and stable bus voltage, especially under varying load conditions, leading to circulating currents and degraded performance. Existing enhancements, such as fixed droop gain adjustments or secondary control schemes, often introduce additional complexity, slower dynamic response, or reliance on centralized communication, which compromise system reliability and scalability. Therefore, there remains a clear need for an adaptive, communication-free droop control strategy that dynamically adjusts parameters in real time, ensuring both precise current sharing under heavy load conditions and effective voltage regulation across diverse operating scenarios in multi-converter DC microgrids.

The following is a summary of the main objectives of this research work:

- Examining the main difficulties associated with DC microgrids’ multiple DC-DC converters for traditional droop control.
- The design and administration for multiple DC-DC converters for standalone applications.
- For converters with equal load current sharing, a straightforward and flexible droop management method is suggested to get rid of circulating current and bus voltage fluctuations.
- To increase load sharing accuracy and mitigate the impact of line resistances, a secondary controller is suggested.

In this research, three buck converters were controlled in this study utilizing adaptive droop control settings, which are monitored and modified live through the principal current sharing loops. This helps to minimize variations in load current sharing. To repair any voltage discrepancies brought on by the droop control technique, an outside addition voltage secondary loop is also employed to restore the proper voltage across the DC microgrid.

## Materials and methods

### Configuration for the multiple buck DC-DC converters configuration scheme

A microgrid is composed of multiple converters connected in parallel that transfer current across several dispersed resources through a single DC bus. Since DC microgrids do not have to worry about frequency, control loop analysis and design may be easier.

The recently brought to light problems and variable load sharing in DC microgrids are examined in this section. Figure 1 depicts a shared changeable load, Droop gain with primary control configuration strategies for multiple buck DC-DC converters. Scheme for configuring multiple buck DC-DC converters, adaptive droop gain with secondary control. A DC-DC buck converter serves as an interface converter between the low-voltage DC bus and the source.

The best power sharing and voltage regulation results are provided by communication-based control systems, including distributed, hierarchical, circular chain, master-slave, and centralized. Nevertheless, they require expensive, failure-prone communication networks that diminish system flexibility, modularity, expandability, and robustness. Therefore, communication-based control methods are more suitable for DC microgrids with compact and fixed structures.

Non-communication-based control methods that rely on locally monitored bus voltages allow DERs to share power. They are inexpensive, easy to install, highly modular, flexible, expandable, and reliable, among other benefits. Therefore, some of the future DC microgrids with many geographically dispersed DERs are better suitable for control methods that do not rely on communication infrastructure. Non-communication-based droop control techniques include conventional droop control, improved droop control, DC bus signals, and mode-adaptive droop control schemes. Providing efficient and appropriate output voltage regulation values for the current shared across the converters is the fundamental goal of DC microgrid control. The effectiveness of traditional droop is significantly impacted by line impedance. There are two modes of operation for the droop control method: linear and non-linear.

Circulating current causes an irregularity in current sharing, which overloads the converters. The system’s efficacy will be diminished by these two consequences. 48 V is the anticipated DC grid voltage level for this project. 48 V is the ideal low-voltage LV DC distribution system voltage, and it is frequently used in the telecom sector. System efficiency is largely determined by the voltage level, which employs 48 V and is the optimum choice for the output low voltage DC transmission system. Table 2 shows the buck DC-DC Converter Parameters. The converters were supplied with two distinct source voltages for examination, and Table 3 shows the buck dc-dc converter operating model parameters in dc microgrids.

**Table 2:** Buck DC-DC converters parameters.

Parameters	Symbol	Values
Resistance of line-1	R_1_	0.2 Ω
Resistance of line-2	R_2_	0.1 Ω
Resistance of line-3	R_**3**_	0.1 Ω
Inductance of line-1	L_c1_	0.2 mH
Inductance of line-2	L_c2_	0.6 mH
Inductance of line-3	L_c**3**_	0.4 mH
Resistance of capacitor 1	$\:{\mathrm{r}}_{\mathrm{c}1}$	0.03 Ω
Resistance of capacitor 2	$\:{\mathrm{r}}_{\mathrm{c}2}$	0.03 Ω
Resistance of capacitor 3	$\:{\mathrm{r}}_{\mathrm{c}3}$	0.03 Ω
Capacitor 1	C_1_	4000 µF
Capacitor 2	C_2_	4000 µF
Capacitor 3	C_3_	4000 µF
Inductance 1	L_1_	0.1 mH
Inductance 2	L_2_	0.1 mH
Inductance 3	L_3_	0.1 mH

**Table 3:** Buck DC-DC converter operating model parameters in DC microgrids.

Operating parameters	Symbol	Values
Input voltage	V_in_	50–80 V
Output voltage	V_out_	48 V
Maximum output current	I_out_	16 A
Switching frequency	F_sw_	100 KHz
Operating power	P_out_	1000 W
Current converter #1 PI	K_P_	0.004
Current converter #1 PI	K_I_	0.2
Voltage converter #1 PI	K_P_	0.02
Voltage converter #1 PI	K_I_	0.2
Voltage restoration converter #1 PI	K_P_	4
Voltage restoration converter #1 PI	K_I_	0.7
Current converter #2 PI	K_P_	0.002
Current converter #2 PI	K_I_	0.2
Voltage Converter #2 PI	K_P_	0.03
Voltage converter #2 PI	K_I_	0.1
Voltage restoration converter #2 PI	K_P_	5
Voltage restoration converter #2 PI	K_I_	0.7
Current converter #3 PI	K_P_	0.001
Current converter #3 PI	K_I_	0.01
Voltage converter #3 PI	K_P_	0.03
Voltage converter #3 PI	K_I_	0.1
Voltage restoration converter #3 PI	K_P_	4
Voltage restoration converter #3 PI	K_I_	0.8

### Formatting of mathematical converters components

**Fig. 2:**
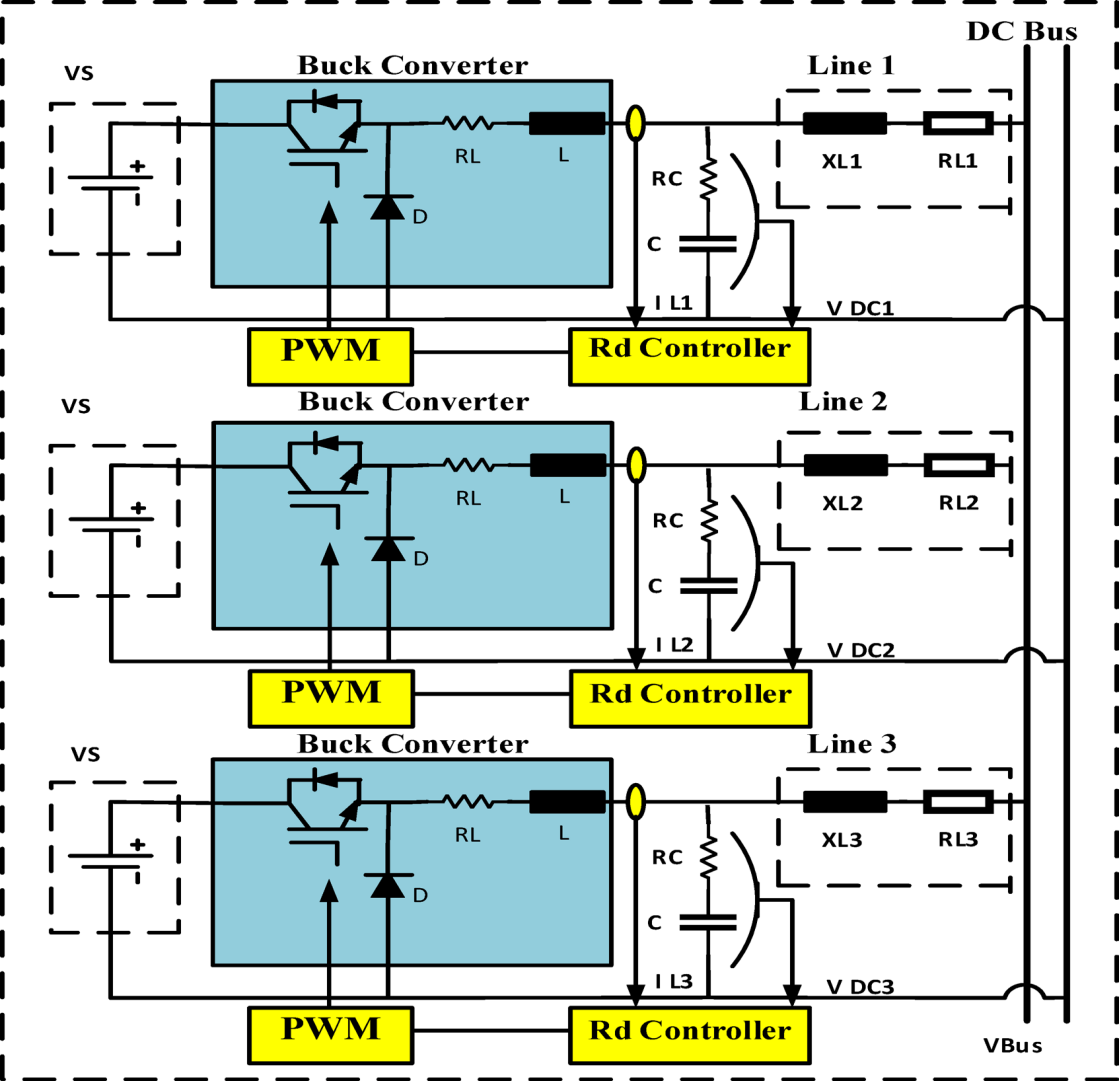
Multiple buck DC-DC converters configuration scheme, droop gain with primary control.

**Fig. 3:**
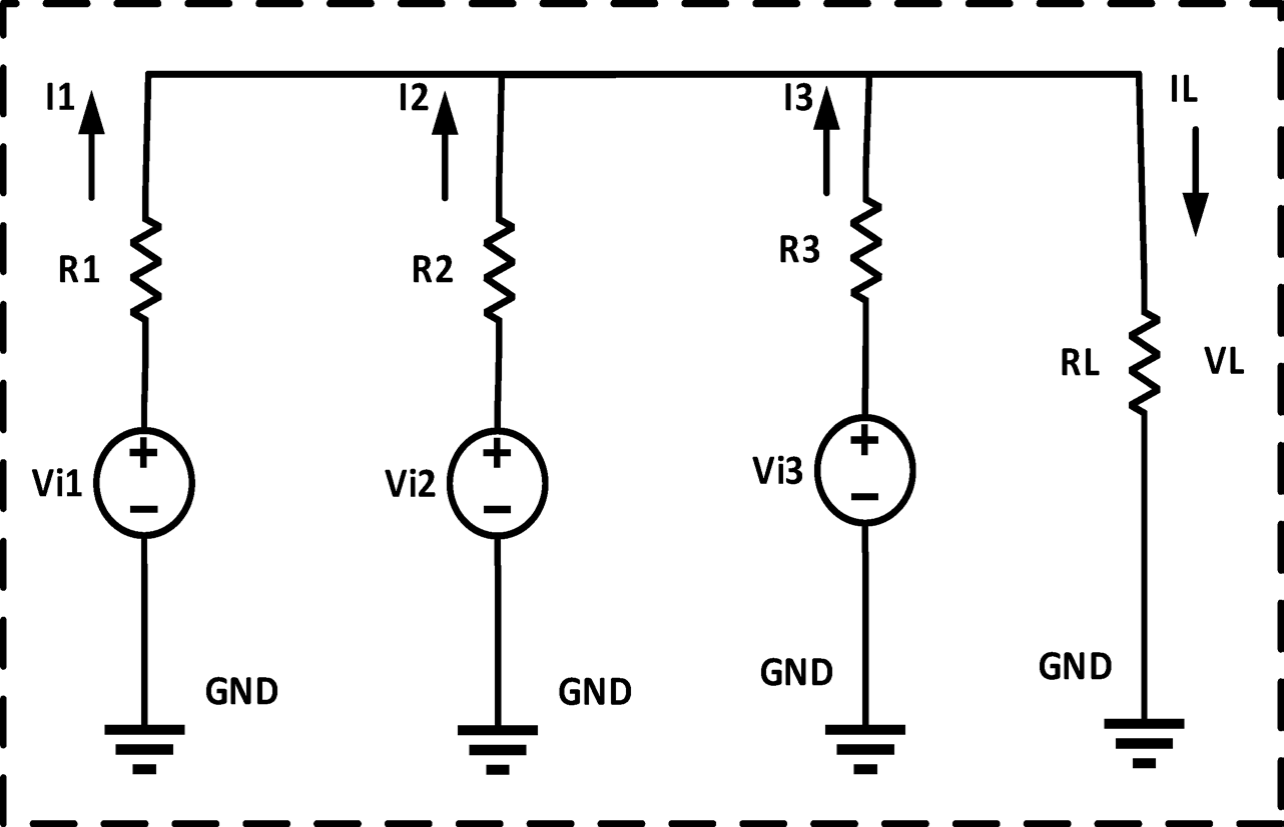
Equivalent DC-DC converters circuit.

**Fig. 4:**
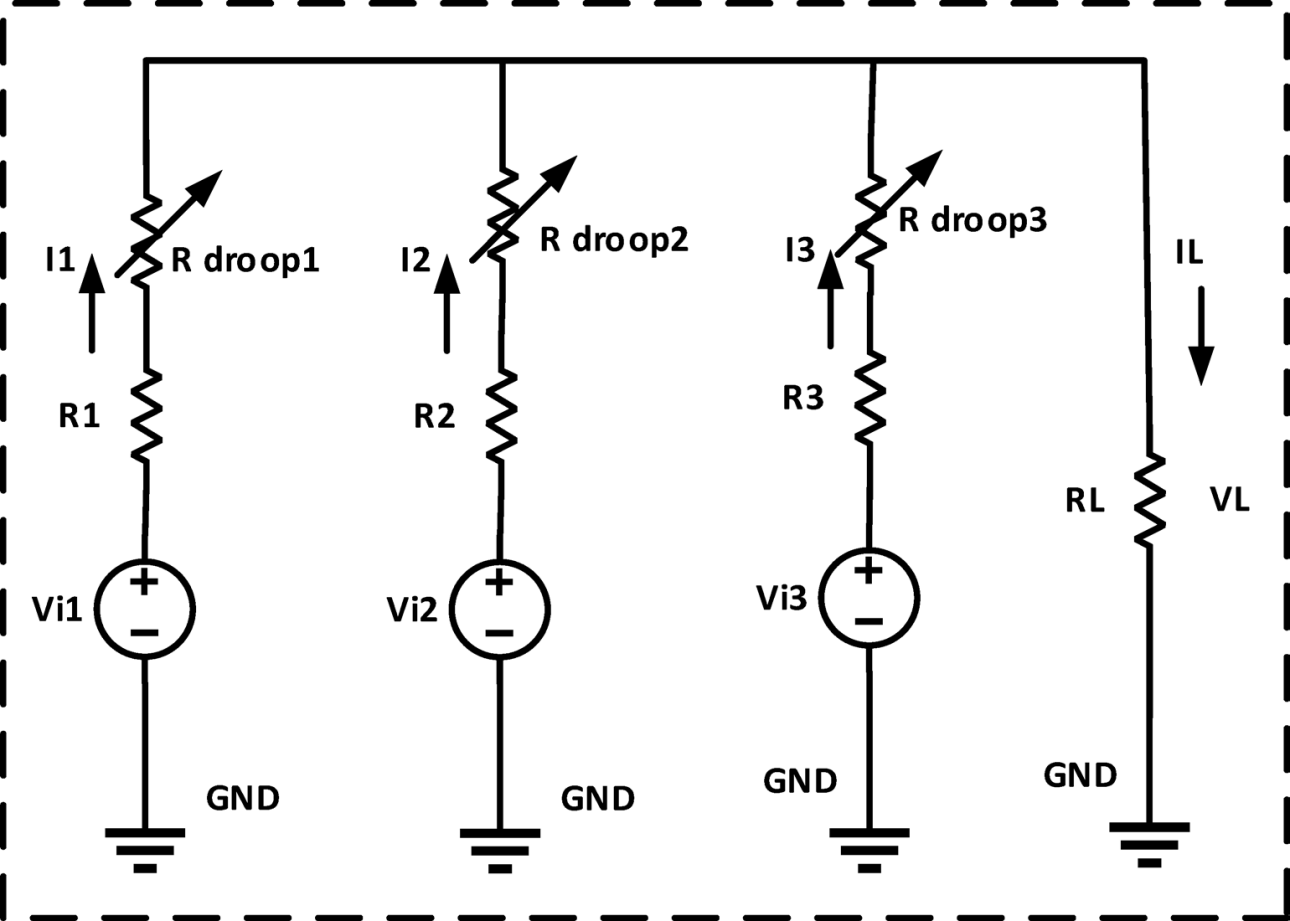
Equivalent DC-DC converters circuit with R droop.

Explains the Fig. 2 schematic for the recommended adaptive control scheme for DC microgrids. The accuracy of current sharing is increased by matching the converter’s nominal voltage. To achieve the exact current sharing error, local control is used to modify the nominal voltage of each converter. Converters with fewer nominal and maximum voltage differences distribute current more evenly. The controller is set up to increase the nominal DC voltage due to the present load sharing and bus voltage fluctuation. Then, using virtual resistance, the reference voltage for every converter is changed to accomplish this. As indicated by R Droop can be changed to alter the reference voltage, power sharing, and R Droop of any converter. The nominal voltage of the converter will be high if the voltage variation is minimal. The low voltage converter will increase its nominal voltage relative to the second, while also reducing the current sharing error. Reducing voltage variation is another function for the secondary loop. A thorough description of each control loop may be found below. The circuits for equivalent DC-DC converters with R_d_ are shown in Figs. 3 and 4^21–24^.

The current output of the converter may be calculated by Eqs. 1, 2, 3.

1 $$\:{\mathrm{I}}_{out\:1}={\mathrm{I}}_{con\:1}-{\mathrm{I}}_{2,\:1}-{\mathrm{I}}_{3,\:1}$$

2 $$\:{\mathrm{I}}_{out\:2}={\mathrm{I}}_{con\:2}-{\mathrm{I}}_{\mathrm{1,2}}-{\mathrm{I}}_{3,\:2}$$

3 $${\mathrm{I}}_{{{\mathrm{out~3}}}} {\text{ = I}}_{{{\mathrm{con~3}}}} {\text{ - I}}_{{{\mathrm{1,~3}}}} {\text{ - I}}_{{{\mathrm{2,~3}}}}$$

4 $$\:{\mathrm{I}}_{out\:1}=\frac{{{\mathrm{V}}_{\mathrm{i}1}({\mathrm{R}}_{\mathrm{L}}{\mathrm{R}}_{2}+{\mathrm{R}}_{2}{\mathrm{R}}_{3}+{\mathrm{R}}_{3}{\mathrm{R}}_{\mathrm{L}})-\left({\mathrm{R}}_{3}{\mathrm{R}}_{\mathrm{L}}\right)\mathrm{V}}_{\mathrm{i}2}-\left({\mathrm{R}}_{2}{\mathrm{R}}_{\mathrm{L}}\right){\mathrm{V}}_{\mathrm{i}3}}{\left({\mathrm{R}}_{1}{\mathrm{R}}_{2}{\mathrm{R}}_{\mathrm{L}}\right)+\left({\mathrm{R}}_{1}{\mathrm{R}}_{2}{\mathrm{R}}_{3}\right)+\left({\mathrm{R}}_{2}{\mathrm{R}}_{3}{\mathrm{R}}_{\mathrm{L}}\right)+\left({\mathrm{R}}_{1}{\mathrm{R}}_{3}{\mathrm{R}}_{\mathrm{L}}\right)}\:\:\:$$

5 $$\:{\mathrm{I}}_{out\:2}=\frac{{{\mathrm{V}}_{\mathrm{i}2}({\mathrm{R}}_{1}{\mathrm{R}}_{3}+{\mathrm{R}}_{1}{\mathrm{R}}_{\mathrm{L}}+{\mathrm{R}}_{3}{\mathrm{R}}_{\mathrm{L}})-\left({\mathrm{R}}_{3}{\mathrm{R}}_{\mathrm{L}}\right)\mathrm{V}}_{\mathrm{i}1}-\left({\mathrm{R}}_{1}{\mathrm{R}}_{\mathrm{L}}\right){\mathrm{V}}_{\mathrm{i}3}}{\left({\mathrm{R}}_{1}{\mathrm{R}}_{2}{\mathrm{R}}_{\mathrm{L}}\right)+\left({\mathrm{R}}_{1}{\mathrm{R}}_{2}{\mathrm{R}}_{3}\right)+\left({\mathrm{R}}_{2}{\mathrm{R}}_{3}{\mathrm{R}}_{\mathrm{L}}\right)+\left({\mathrm{R}}_{1}{\mathrm{R}}_{3}{\mathrm{R}}_{\mathrm{L}}\right)}\:\:\:$$

6 $$\:{\mathrm{I}}_{out\:3}=\frac{{{\mathrm{V}}_{\mathrm{i}3}({\mathrm{R}}_{1}{\mathrm{R}}_{2}+{\mathrm{R}}_{2}{\mathrm{R}}_{\mathrm{L}}+{\mathrm{R}}_{1}{\mathrm{R}}_{\mathrm{L}})-\left({\mathrm{R}}_{2}{\mathrm{R}}_{\mathrm{L}}\right)\mathrm{V}}_{\mathrm{i}1}-\left({\mathrm{R}}_{1}{\mathrm{R}}_{\mathrm{L}}\right){\mathrm{V}}_{\mathrm{i}3}}{\left({\mathrm{R}}_{1}{\mathrm{R}}_{2}{\mathrm{R}}_{\mathrm{L}}\right)+\left({\mathrm{R}}_{1}{\mathrm{R}}_{2}{\mathrm{R}}_{3}\right)+\left({\mathrm{R}}_{2}{\mathrm{R}}_{3}{\mathrm{R}}_{\mathrm{L}}\right)+\left({\mathrm{R}}_{1}{\mathrm{R}}_{3}{\mathrm{R}}_{\mathrm{L}}\right)}\:\:\:$$

#### Mathematical component formatting

However, this is not possible in performance. Each converter’s reference voltage can be changed to accomplish the required load sharing. This is accomplished by altering each converter’s reference voltage using a fictitious resistance called R Droop. The matching equations can be expressed as follows.

7 $$\:{{\mathrm{V}}_{\mathrm{D}\mathrm{C}\:\mathrm{n}\mathrm{e}\mathrm{w}}=\mathrm{V}}_{1\:\mathrm{r}\mathrm{e}\mathrm{f}}={\mathrm{I}}_{1}\left({\mathrm{R}}_{1}+{\mathrm{R}}_{\mathrm{d}1}\right)+{\mathrm{R}}_{\mathrm{L}\:}{\mathrm{I}}_{\mathrm{L}\:}$$

Equations 7, 8 and 9 can be used to demonstrate how altering the R_d_ value can alter the reference voltage.

8 $$\:{{\mathrm{V}}_{\mathrm{D}\mathrm{C}\:\mathrm{n}\mathrm{e}\mathrm{w}}=\mathrm{V}}_{2\:\mathrm{r}\mathrm{e}\mathrm{f}}={\mathrm{I}}_{2}\left({\mathrm{R}}_{2}+{\mathrm{R}}_{\mathrm{d}2}\right)+{\mathrm{R}}_{\mathrm{L}\:}{\mathrm{I}}_{\mathrm{L}\:}$$

9 $$\:{{\mathrm{V}}_{\mathrm{D}\mathrm{C}\:\mathrm{n}\mathrm{e}\mathrm{w}}=\mathrm{V}}_{3\:\mathrm{r}\mathrm{e}\mathrm{f}}={\mathrm{I}}_{3}\left({\mathrm{R}}_{3}+{\mathrm{R}}_{\mathrm{d}3}\right)+{\mathrm{R}}_{\mathrm{L}\:}{\mathrm{I}}_{\mathrm{L}\:}$$

According to (10), the converter’s updated voltage reference in droop control is as follows.

10 $$\:{V}^{*}={\mathrm{V}}_{\mathrm{r}\mathrm{e}\mathrm{f}}-{\mathrm{R}}_{\mathrm{d}}\:\mathrm{*}\mathrm{I}\:$$

Equation 11 require that the droop settings be adjusted to control the source converters and increase the bus voltage such that each converter’s output voltage is the same^4^.

11 $$\:{\mathrm{V}}^{\mathrm{*}}={\mathrm{V}}_{\mathrm{r}\mathrm{e}\mathrm{f}}-{(\mathrm{R}}_{\mathrm{d}}\:\pm\:{\Delta\:}\mathrm{R})\mathrm{*}\mathrm{I}\:$$

Primary Droop Law

12 $$\:{\mathrm{V}}_{\mathrm{i}}={\mathrm{V}}_{\mathrm{r}\mathrm{e}\mathrm{f}}-{\mathrm{R}}_{\mathrm{d}}\:\:{\mathrm{I}}_{\mathrm{i}}$$

Control Law of the Secondary Voltage Loop

The secondary loop measures the bus voltage error:

13 $$\:{\mathrm{e}}_{\mathrm{v}}\:\left(\mathrm{t}\right)={\mathrm{V}}_{\mathrm{r}\mathrm{e}\mathrm{f}}-{\mathrm{V}}_{\mathrm{b}\mathrm{u}\mathrm{s}\:}\left(\mathrm{t}\right)$$

Mathematical Implementation of Droop Shift

14 $$\:{\mathrm{V}}_{\mathrm{i}}={\mathrm{V}}_{\mathrm{r}\mathrm{e}\mathrm{f}}-{\mathrm{R}}_{\mathrm{d}}\:\:{\mathrm{I}}_{\mathrm{i}}+\varDelta\:\mathrm{V}$$

To ensure appropriate load sharing across all converters in DC microgrids, the primary loop is employed to continually update the virtual resistances value from the previous section. In addition to any defects in the current or voltage feedback, the load also affects the deviation of the bus voltage^10^.

#### The primary loops

**Fig. 5:**
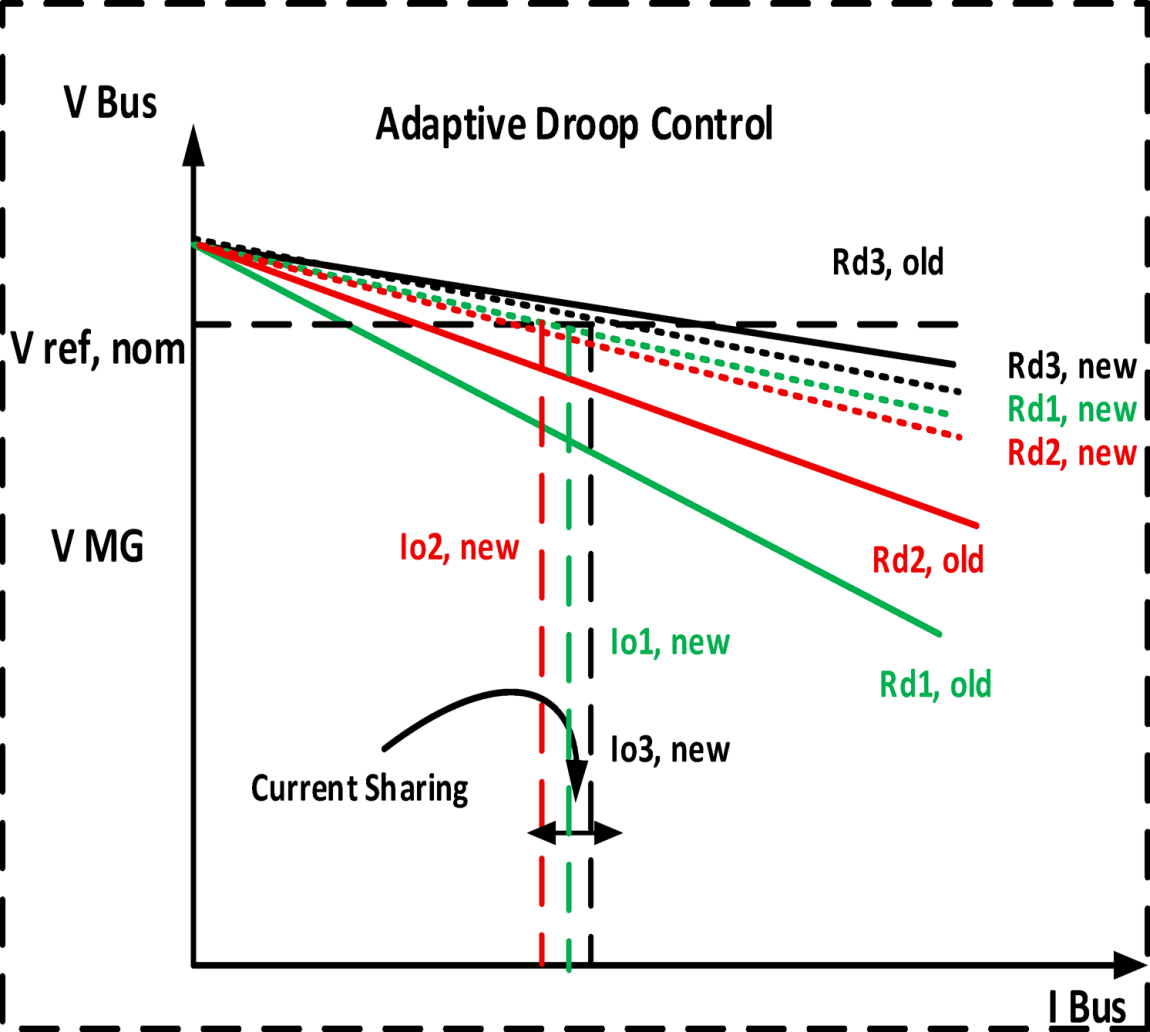
The relationship between current sharing and adaptive droop gain.

**Fig. 6:**
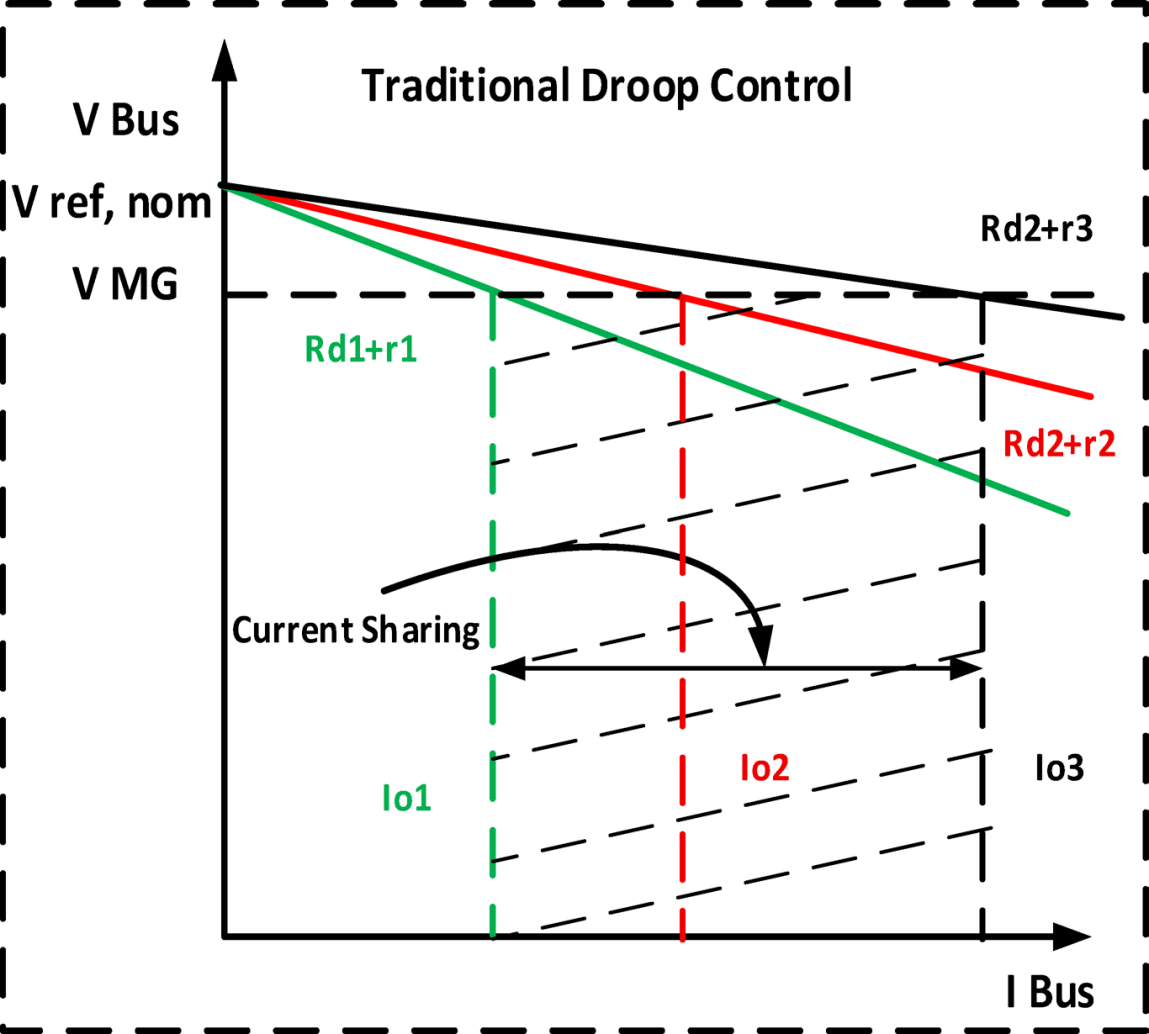
The relationship between current sharing and constant virtual droop resistances.

The primary purpose for all converters in DC microgrids is to ensure proper load sharing. The proposed adaptive droop concept is discussed using Fig. 5. Where Rd1, Rd2 and Rd3 are the initial droop characteristic lines by using Fig. 6. The relationship between current sharing and constant virtual droop resistances, with non-desired current sharing I1, I2 and I3, and VMG is the bus voltage deviation. Adaptive control is used to relocate the droop characteristic lines to a point that meets the DC microgrid control criterion. Each movement in this work can be presented separately using two distinct stages. The initial phase is control of the adaptive current sharing. ± ΔR is added to the traditional droop Eq. (11) to update the value of virtual resistance and improve power sharing. Figure 5 illustrates a flowchart of the suggested strategy’s steps.

## Results

**Fig. 7:**
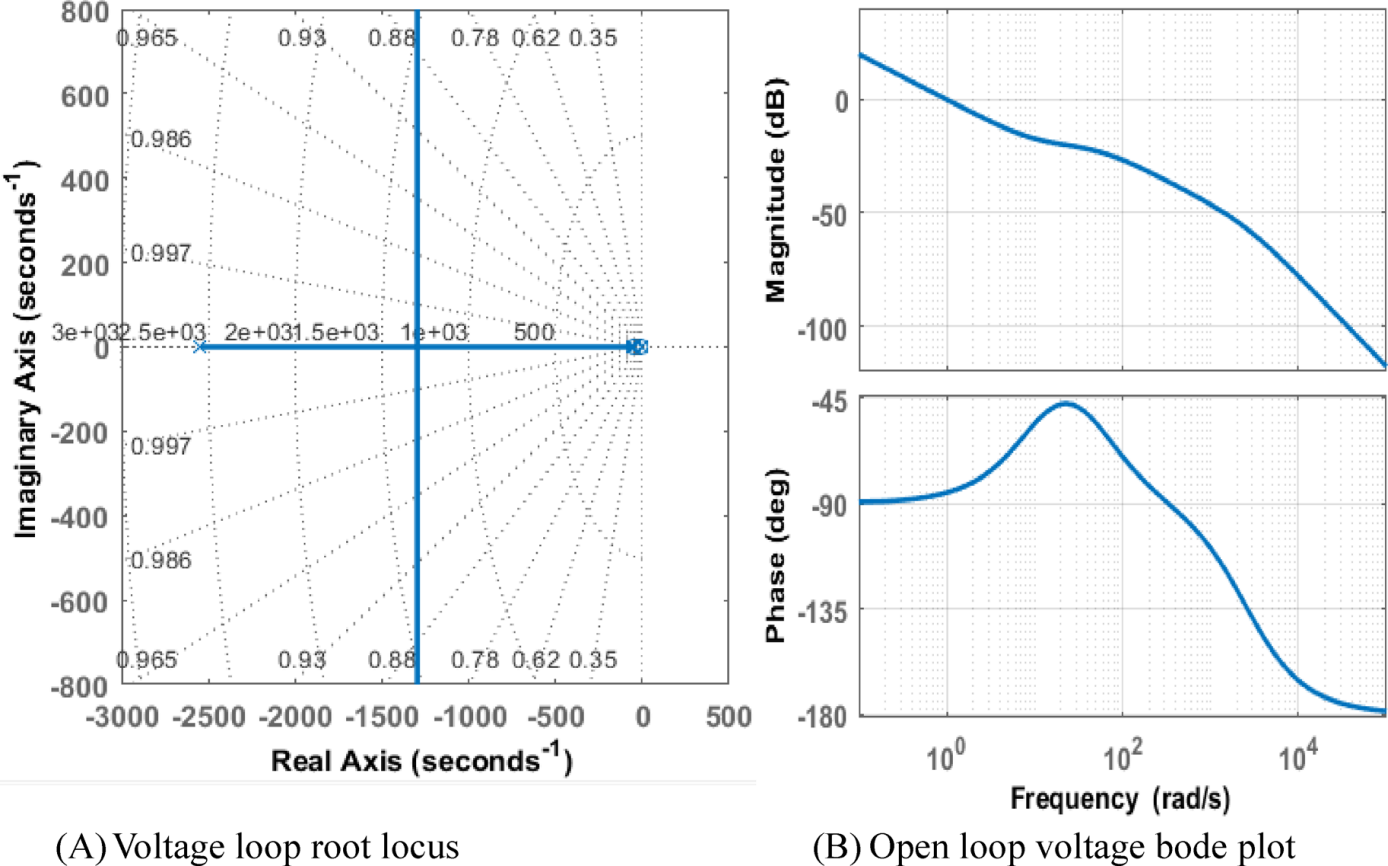
Root locus and bode plot (**A**) voltage loop root locus (**B**) open loop voltage bode plot.

**Fig. 8:**
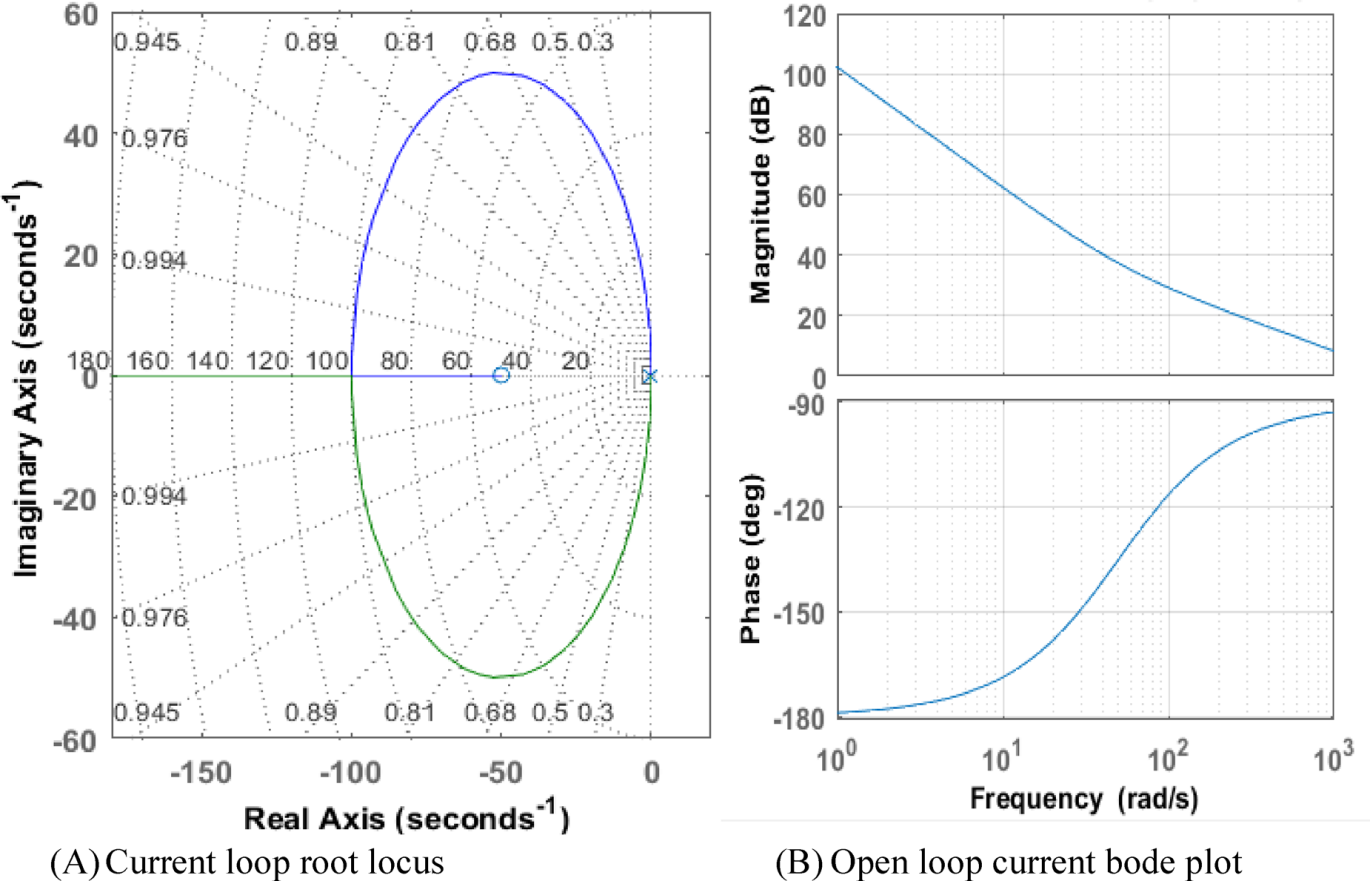
Root locus and bode plot (**A**) current loop root locus (**B**) open loop current bode plot.

Although they reflect separate domains and characteristics of the system, root locus and Bode plots are complementing techniques used in control systems to study system stability, as seen in the Figs. 7 and 8. The Bode plot is used for stability analysis through gain and phase margins and examines system behavior in the frequency domain, demonstrating how magnitude and phase change with frequency. use phase margin and gain margin to determine stability. evaluating the system’s adaptability to modifications. giving a simple explanation of a system’s frequency response.

By mapping the trajectories of closed-loop poles as a parameter like gain changes, the root locus studies system behavior in the time domain. It is used to build controllers and comprehend time-domain reactions like damping and overshooting. examining the stability of the system as the gain varies. Demonstrating how the addition of poles or zeros influences system behavior while designing controllers. indirectly revealing details on the time-domain response of the system, including oscillation frequency and damping.

Tables 4, 5, and 6 give the simulation results for the load sharing error and circulation current for various cable resistance conditions, varying loads and cable resistances, and varying source voltages and loads.

**Table 4:** comparing two strategies with a change in load resistance 9 Ω.

Method	V bus (V)	I bus (I1, I2, I3) (A)	$\:\boldsymbol{\Delta\:}$ V Bus %	$\:\boldsymbol{\Delta\:}$ I circulate %
Droop gain with primary control	47.2	5.245 (1.471, 1.882, 1.892)	1.67	16.05
Adaptive droop gain with secondary control	47.97	5.328 (1.779, 1.756, 1.793)	0.062	1.39

**Table 5:** comparing two strategies with a change in load resistance 4.5 Ω.

Method	V bus (V)	I bus (I1, I2, I3) (A)	$\:\boldsymbol{\Delta\:}$ V bus %	$\:\boldsymbol{\Delta\:}$ I circulate %
Droop gain with primary control	46.75	10.382 (2.856, 3.661, 3.865)	2.604	19.43
Adaptive droop gain with secondary control	47.92	10.651 (3.512, 3.574, 3.565)	0.167	1.16

In these cases, there is a variation in the value of current sharing between source converters. When loading is light, medium, or heavy, the droop control with primary control has the largest current sharing error of 16.05%, 19.43%, and 25.05%, whereas the recommended adaptive droop with secondary control has the highest current sharing error of 1.39%, 1.16%, and 0.53%. According to the results, the present sharing error is comparatively low under various loading scenarios. Additionally, the suggested adaptive droop with secondary control provides a voltage deviation of 0.062%, 0.167%, and 0.208%, while the proposed technique permits a voltage variation of 1.67%, 2.604%, and 3.23% for droop control with main control. should stay within reasonable parameters even in a range of low, medium, and high loading conditions.

**Table 6:** comparing two strategies with a change in load resistance 3.27 Ω.

Method	V bus (V)	I bus (I1, I2, I3) (A)	$\:\boldsymbol{\Delta\:}$ V bus %	$\:\boldsymbol{\Delta\:}$ I circulate %
Droop gain with primary control	46.45	14.183 (3.547, 5.312, 5.324)	3.23	25.05
Adaptive droop gain with secondary control	47.9	14.643 (4.859, 4.886, 4.898)	0.208	0.53

The online selection of the droop coefficients in this study has improved the conventional droop control. Droop-controlled DC microgrids can increase the accuracy of current sharing and decrease bus voltage fluctuation. When voltage and current fluctuate, the online adaptation technique is intended to adjust the droop control resistance. There is no need for inter-source converter communication or extra measurements with the suggested control because it is straightforward.

**Fig. 9:**
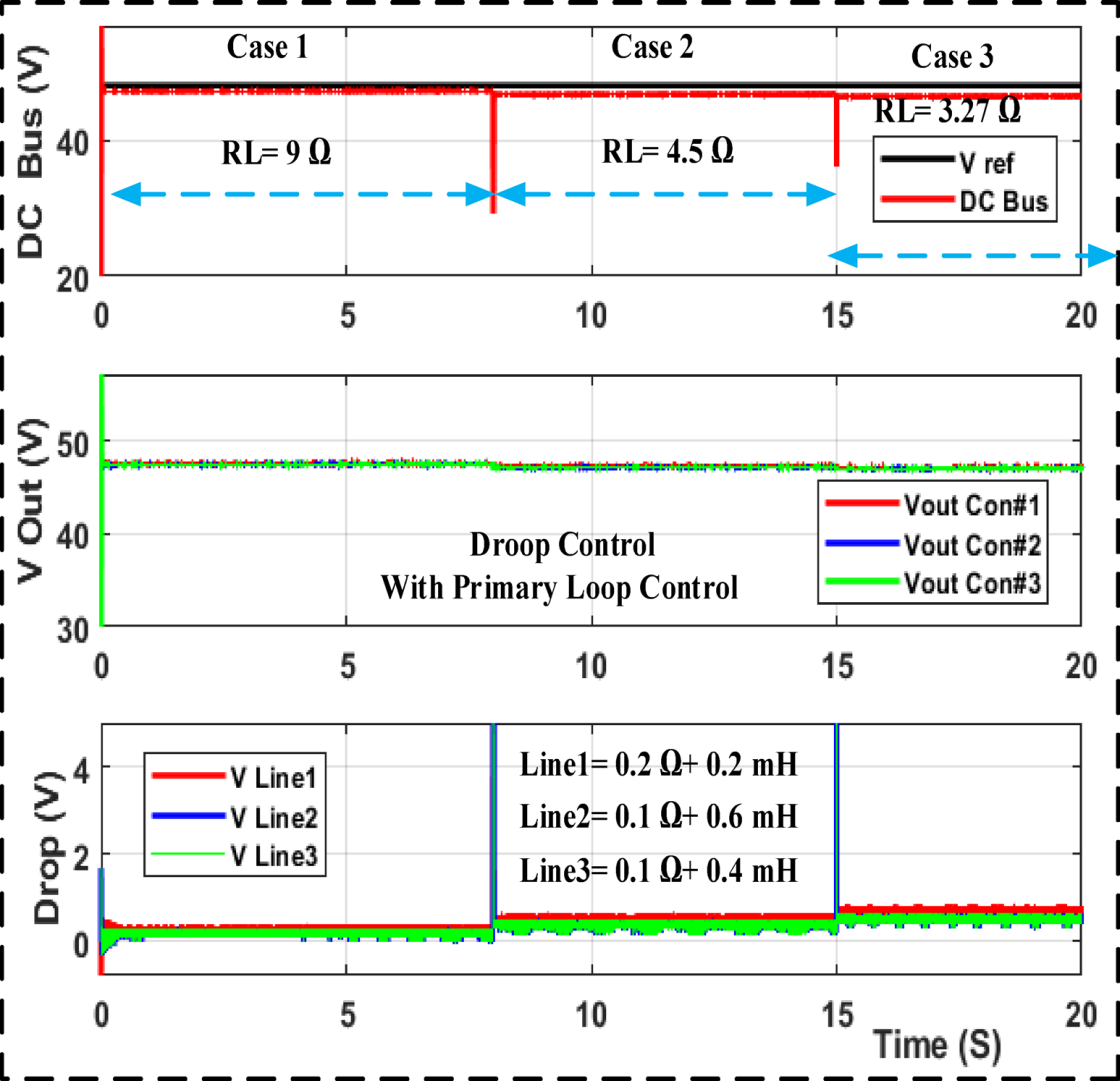
Output voltage and drop voltage The MATLAB/SIMULINK transient response for droop gain with primary control.

**Fig. 10:**
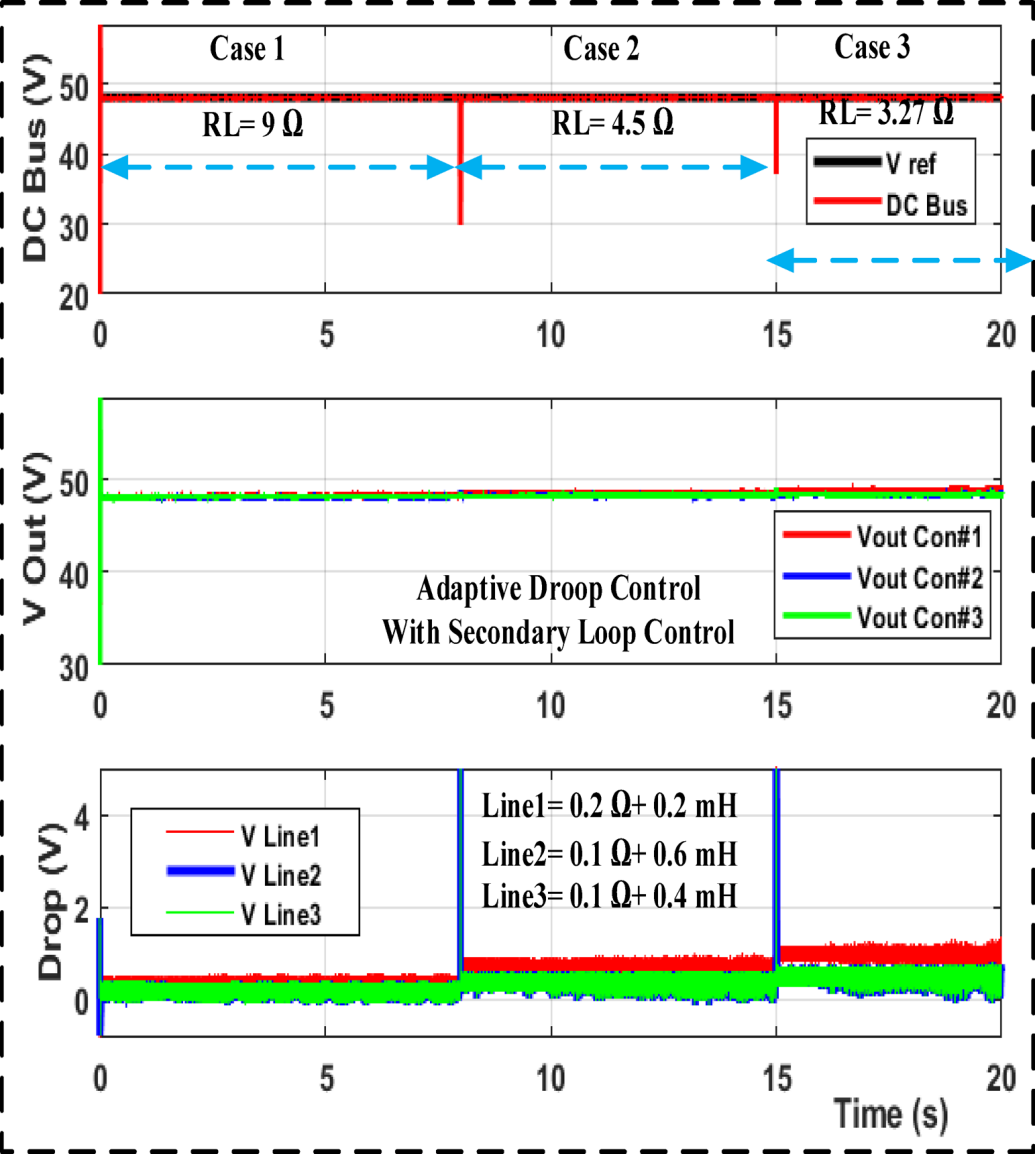
Output voltage and drop voltage the MATLAB/SIMULINK transient response for adaptive droop gain with secondary control.

With a step load shift from 9 to 4.5 and 3.27 ohm and a step change in the input voltage using MATLAB/SIMULINK, Fig. 9 shows the output voltage and drop line for cables responding to droop gain with primary control. Figure 10 shows the results of the adaptive droop gain with secondary control MATLAB/SIMULINK, even though the current sharing error is significantly reduced. The voltage variation occurs under different operating situations. Despite this, they share the same load current and avoid bus voltage variance among converters.

**Fig. 11:**
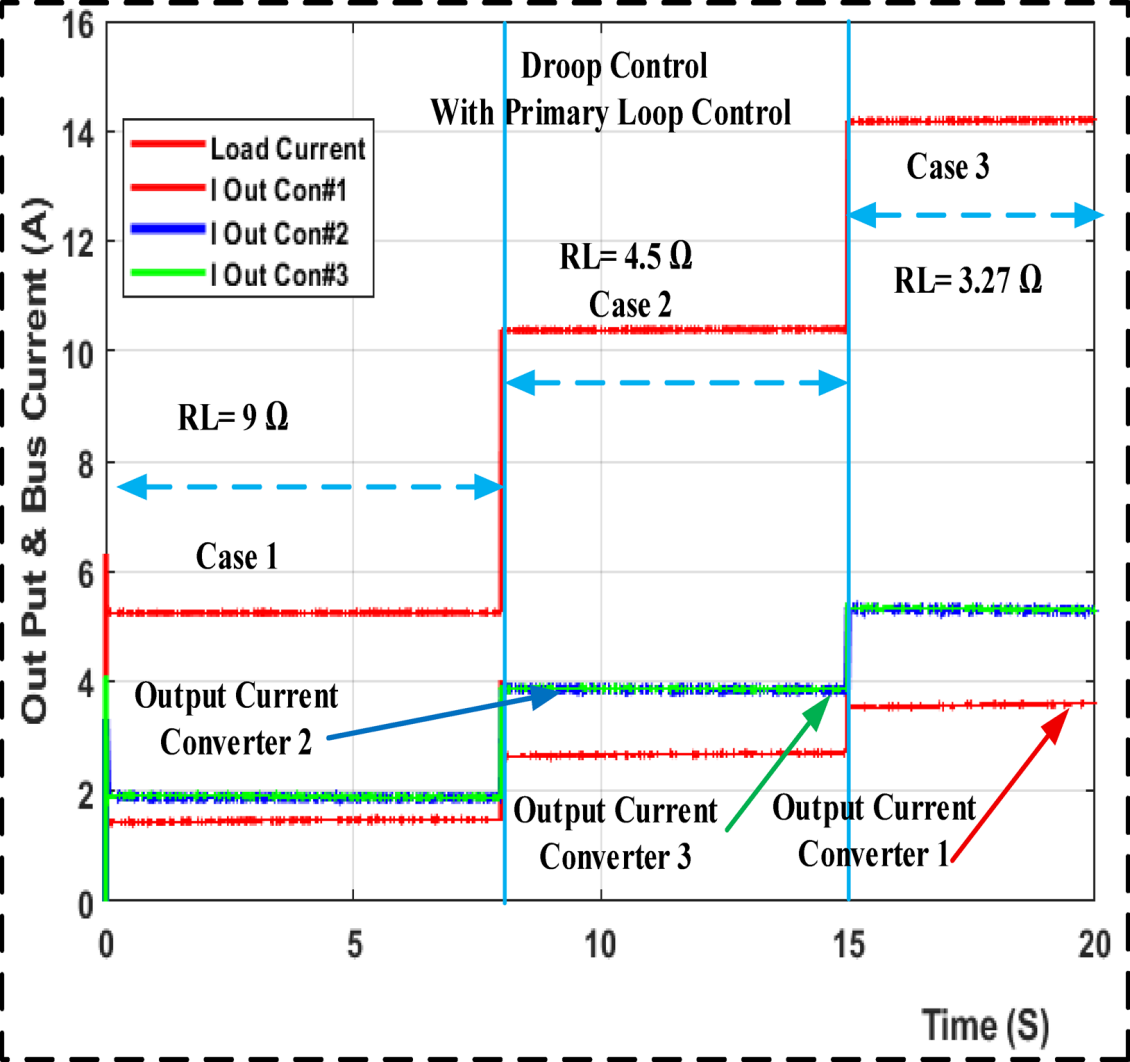
Output loads current the MATLAB/SIMULINK transient response for droop gain with primary control.

**Fig. 12:**
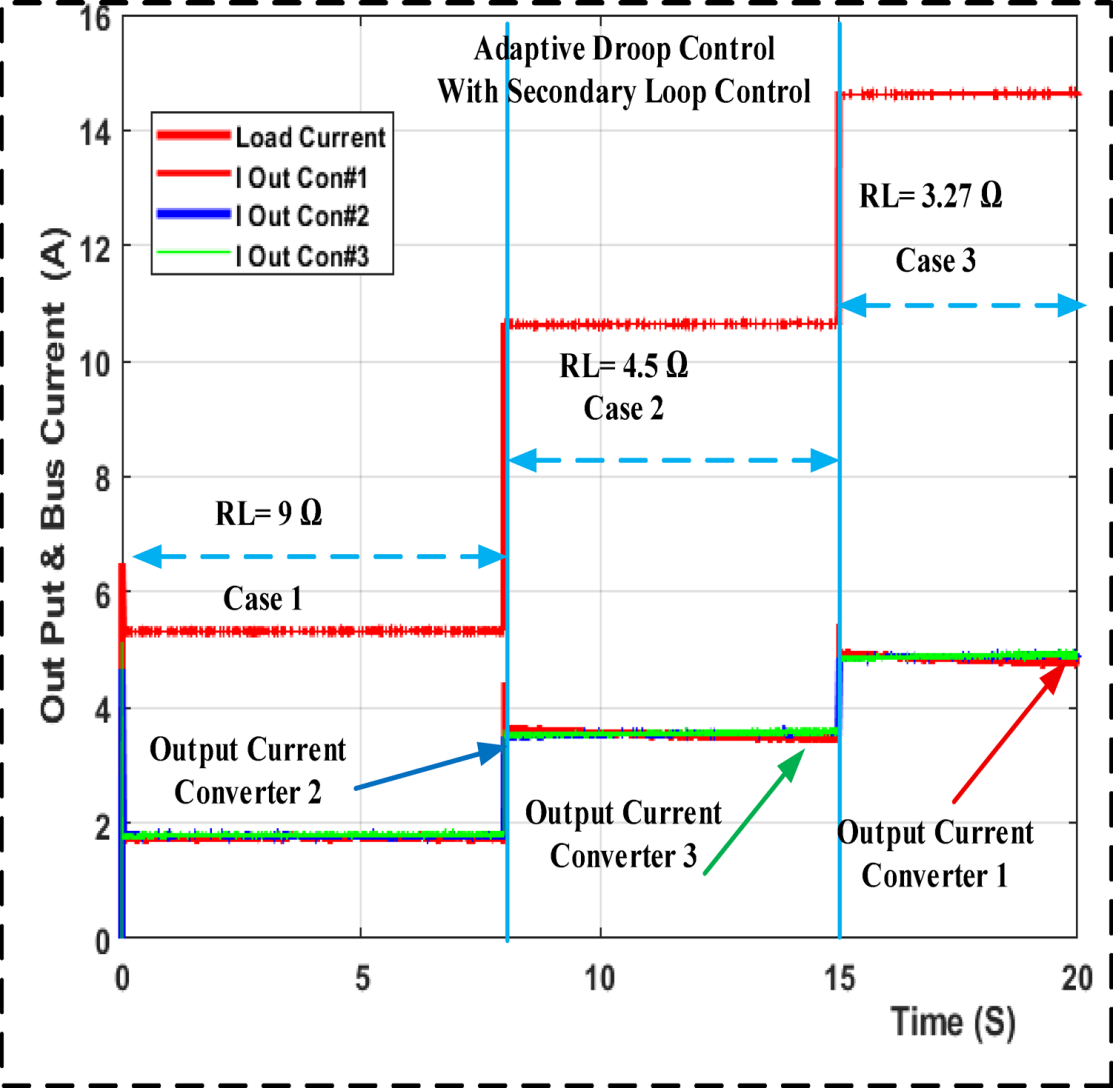
Output loads current the MATLAB/SIMULINK transient response for adaptive droop gain with secondary control.

The output load current response for droop gain with primary control is shown in Fig. 11 along with a step change in the input voltage using MATLAB/SIMULINK and a step change in the load from 9 to 4.5 and 3.27 ohm. Figure 12 shows the results of the adaptive droop gain with secondary control MATLAB/SIMULINK, even though the current sharing error is significantly reduced. The voltage variation occurs under different operating situations. Even so, they share the same load current while removing the circulated current across converters.

## Comparison discussion

The simulation results clearly demonstrate the significant drawbacks of conventional fixed droop control in DC microgrids, primarily the issue of circulating currents leading to poor current sharing and voltage deviation. As shown in the comparative analysis, fixed droop control resulted in substantial current sharing errors ranging from 16.05% to 25.05% across different load conditions, with corresponding bus voltage deviations of 1.67% to 3.23%. These errors stem from the system’s inability to account for disparities in line resistances and converter parameters, causing some converters to be overloaded while others are underutilized. This inefficient current distribution not only diminishes the system’s overall efficacy but also potentially stresses individual converters, reducing their operational lifespan and compromising the microgrid’s reliability.

**Fig. 13:**
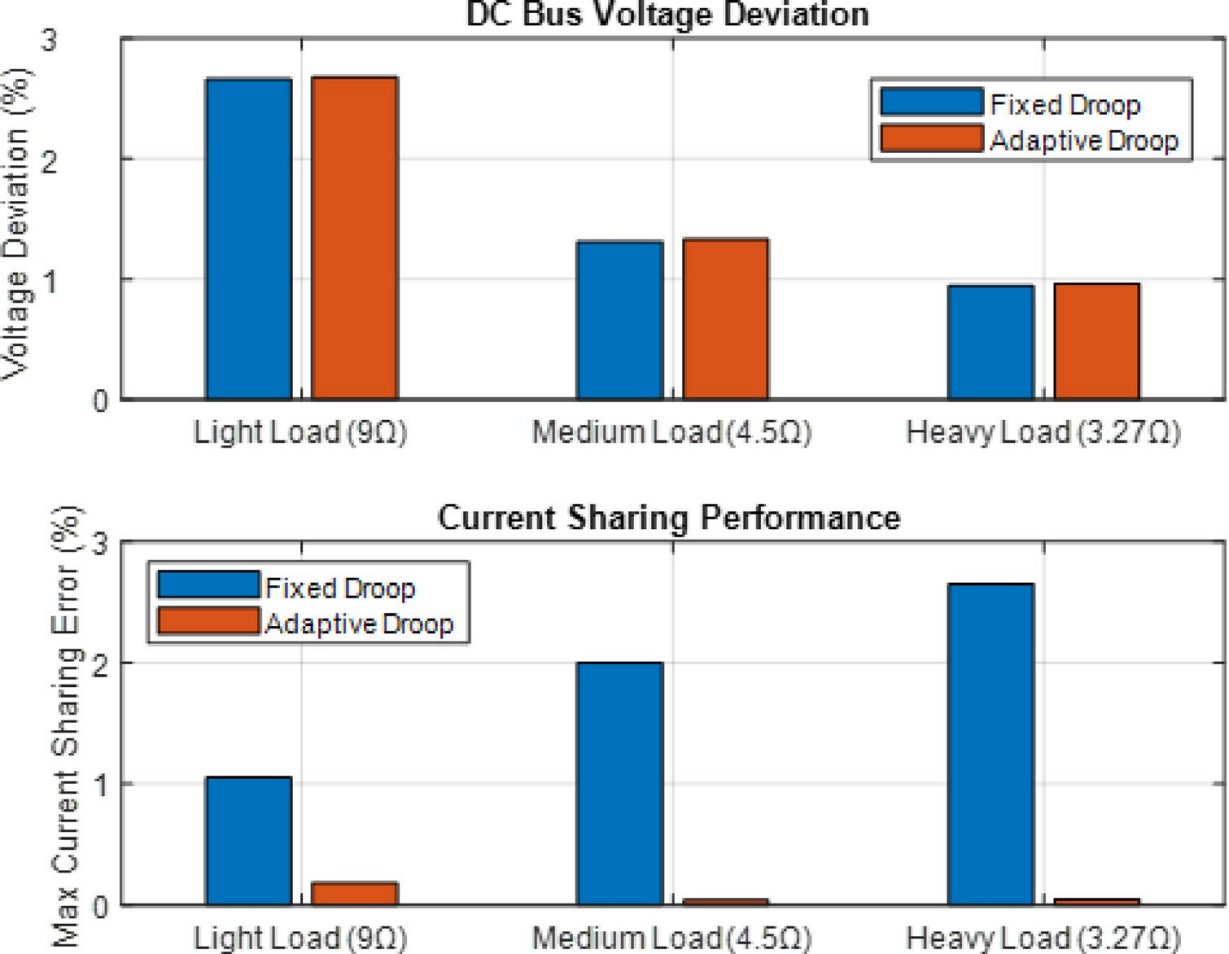
Comparison of fixed droop control. Adaptive droop control in a DC microgrid.

**Fig. 14:**
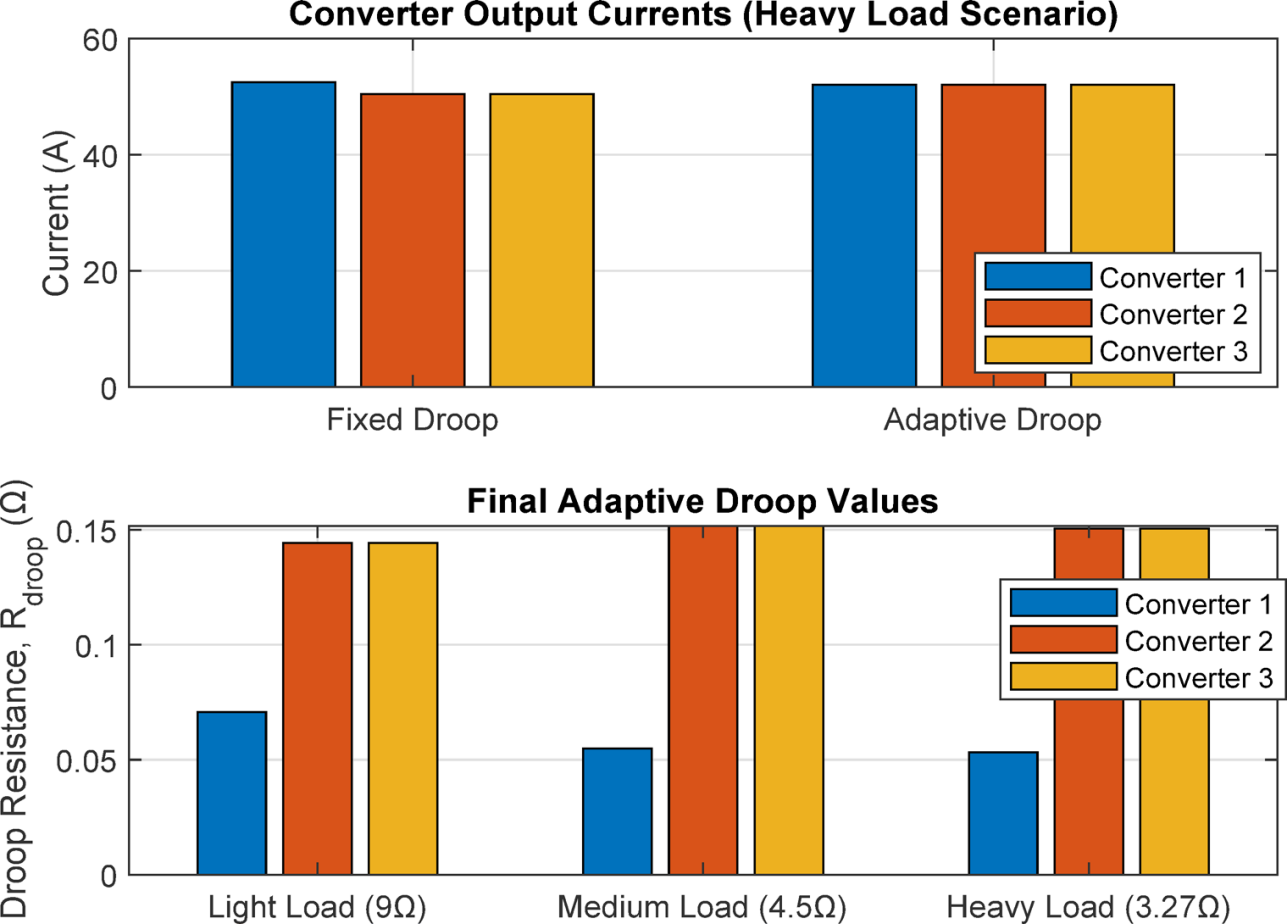
(**a**) converter output currents (heavy load scenario) (**b**) final adaptive droop values.

In contrast, the implementation of the proposed adaptive droop control strategy effectively mitigates these issues by dynamically adjusting the virtual droop resistances based on real-time current measurements. The adaptive approach dramatically reduced current sharing errors to below 1.39% across all load scenarios while maintaining bus voltage deviation within 0.208%. This performance enhancement is achieved through the controller’s ability to autonomously compensate for system imbalances without requiring communication between converters. By optimizing the droop coefficients online, adaptive control ensures precise current sharing while minimizing voltage regulation errors, thereby increasing the system’s overall efficiency, stability, and resilience to varying load conditions and component mismatches.by using Fig. 13. Comparison of Fixed droop control. Adaptive Droop Control in a DC Microgrid and Fig. 14. (a) Converter Output Currents (Heavy Load Scenario (b) Final Adaptive Droop Values.

The MATLAB simulation results, illustrated in the accompanying figure, provide compelling visual evidence for the superiority of the adaptive droop control strategy. The plotted comparisons clearly show that the adaptive method (represented in the bar charts) consistently outperforms the fixed droop approach across all tested load scenarios. Specifically, the figure demonstrates a drastic reduction in both DC bus voltage deviation and maximum current sharing error when the adaptive controller is employed. Furthermore, the visualization of converter currents in the heavy load condition reveals a significantly more balanced current distribution among the three converters, effectively mitigating the circulating currents that plague the fixed droop system. Finally, the plot of the final adaptive droop values illustrates how the controller intelligently and autonomously adjusts the virtual resistance for each converter to achieve this optimal performance, dynamically compensating for the inherent imbalances in the system’s line resistances.

**Fig. 15:**
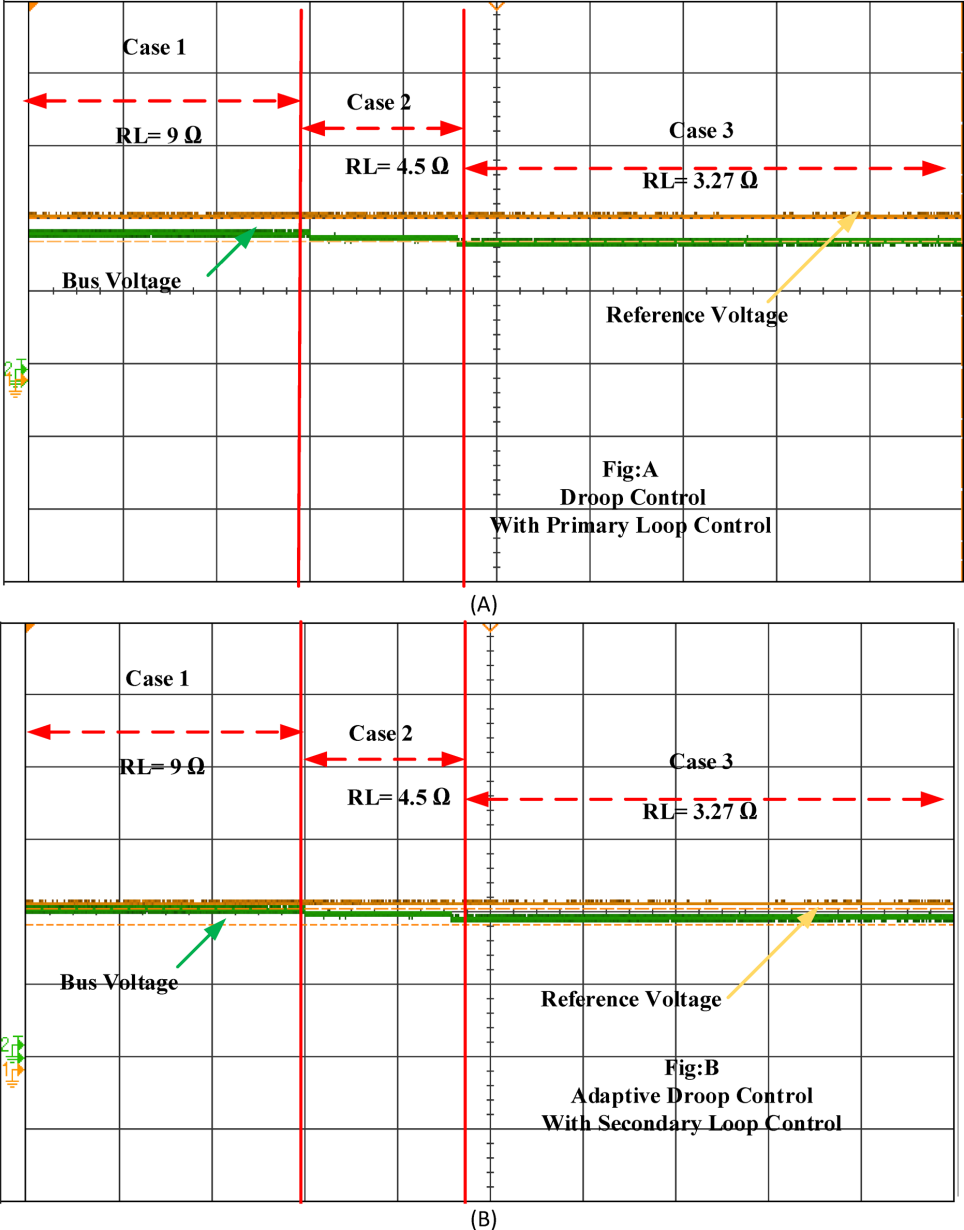
Bus voltage is compared at (**A**) droop gain with primary control and (**B**) adaptive droop gain with secondary control when input voltage changes and load varies from 9 Ω to 4.5 Ω and 3.27 Ω.

As a result, Real-Time Simulation OPAL-RT is a great help in the development and assessment of the suggested adaptive controller for its performance when the suggested algorithm is tested using a range of bus voltages at (A) Droop gain with primary control and (B) Adaptive droop gain with secondary control when input voltage varies and load varies from 9 Ω to 4.5 Ω and 3.27 Ω as shown in the Fig. 15.

## Conclusion

The major current sharing loops are used to check and update the adaptive droop control parameters, which helps to lessen variations in load current sharing. To repair any voltage discrepancies brought on by the droop control methodology, an outside addition voltage secondary loop is also employed to restore the correct voltage level across the DC microgrid.

- In the microgrid, load sharing between the converters is accomplished by the droop control technique.
- By eliminating the bus voltage deviation, the suggested adaptive technique increases the accuracy of current sharing in DCMGs with droop control.
- There are two suggested loops: the first improves current sharing, while the second keeps the bus voltage at its purported level. Without requiring any measurements or communication channels between the source converters, the suggested control approach is straightforward.
- The control methodology is put to the test and assessed in various operating environments. To confirm that the proposed method is effective, the effect of the line impedance on the three converters is taken into consideration.
- To summarize, this new adaptive droop control system uses nested control loops and dynamically modifies control parameters to enhance load current sharing and voltage regulation in DC microgrids. To remedy any voltage discrepancies brought on by the droop control technique, a secondary loop is also utilized to restore the correct voltage level across the DC microgrid.

## Data Availability

The datasets produced during these simulations including voltage trajectories, current sharing profiles, controller response logs, and system state variables are not stored in a public repository. However, they are available from the authors upon reasonable request.To access the data, interested researchers may: 1. Request MATLAB/Simulink model files used for microgrid modeling and adaptive-control testing. 2. Request RTS output files generated using the Real-Time Simulation Fundamentals platform (e.g., OPAL-RT, Typhoon HIL, or comparable systems, depending on the experiment)0. 3. Contact the corresponding author to obtain the datasets and documentation describing the simulation setup, parameters, and procedures for reproduction.
